# 10-Ethyl-3-(5-methyl-1,3,4-oxadiazol-2-yl)-10*H*-phenothia­zine

**DOI:** 10.1107/S160053681200462X

**Published:** 2012-02-10

**Authors:** Yu-Zhen Pan, You-Gui Wang, Jian-Hui Liu, Li-Cheng Sun

**Affiliations:** a College of Chemistry, Dalian University of Technology, 116024 Dalian, Liaoning, People’s Republic of China; b State Key Laboratory of Fine Chemicals, DUT-KTH Joint Education and Research Center on Molecular Devices, Dalian University of Technology, 116024 Dalian, Liaoning, People’s Republic of China; c Department of Chemistry, School of Chemical Science and Engineering, KTH Royal Institute of Technology, Stockholm 10044, Sweden

## Abstract

In the title compound, C_17_H_15_N_3_OS, the phenothia­zine ring system is slightly bent, with a dihedral angle of 13.68 (7)° between the benzene rings. The dihedral angle between the oxadiazole ring and the adjacent benzene ring is 7.72 (7)°. In the crystal, a π–π inter­action with a centroid–centroid distance of 3.752 (2) Å is observed between the benzene rings of neighbouring mol­ecules.

## Related literature

For general background to phenothia­zine derivatives, see: Kim *et al.* (2011 ▶); Hagfeldt *et al.* (2010 ▶). For related structures, see: Chu & Van der Helm (1975 ▶); Hdii *et al.* (1998 ▶); Li, Hu *et al.* (2009 ▶); Li, Lv *et al.* (2009 ▶); Yu *et al.* (2011 ▶).
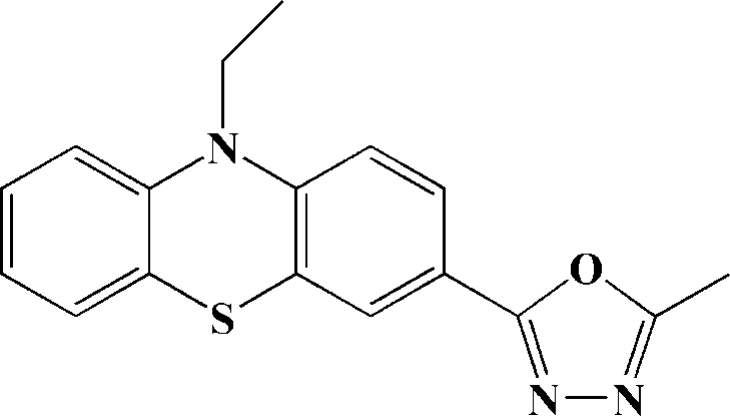


## Experimental

### 

#### Crystal data

C_17_H_15_N_3_OS
*M* *_r_* = 309.38
Triclinic, 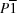

*a* = 7.6752 (4) Å
*b* = 8.2913 (4) Å
*c* = 12.9469 (8) Å
α = 84.870 (4)°
β = 82.569 (4)°
γ = 63.696 (3)°
*V* = 731.92 (7) Å^3^
*Z* = 2
Mo *K*α radiation
μ = 0.23 mm^−1^
*T* = 293 K
0.15 × 0.15 × 0.10 mm

#### Data collection

Bruker SMART APEX diffractometer
Absorption correction: multi-scan (*SADABS*; Sheldrick, 2004 ▶) *T*_min_ = 0.968, *T*_max_ = 0.979
5348 measured reflections
2554 independent reflections
2217 reflections with *I* > 2σ(*I*)
*R*_int_ = 0.020

#### Refinement

*R*[*F* ^2^ > 2σ(*F* ^2^)] = 0.039
*wR*(*F* ^2^) = 0.107
*S* = 1.05
2554 reflections
201 parameters
H-atom parameters constrained
Δρ_max_ = 0.33 e Å^−3^
Δρ_min_ = −0.28 e Å^−3^

### 

Data collection: *SMART* (Bruker, 2001 ▶); cell refinement: *SAINT* (Bruker, 2001 ▶); data reduction: *SAINT*; program(s) used to solve structure: *SHELXTL* (Sheldrick, 2008 ▶); program(s) used to refine structure: *SHELXTL*; molecular graphics: *DIAMOND* (Brandenburg & Putz, 2004 ▶) and *SHELXTL*; software used to prepare material for publication: *SHELXTL*.

## Supplementary Material

Crystal structure: contains datablock(s) I, global. DOI: 10.1107/S160053681200462X/is5065sup1.cif

Structure factors: contains datablock(s) I. DOI: 10.1107/S160053681200462X/is5065Isup3.hkl

Supplementary material file. DOI: 10.1107/S160053681200462X/is5065Isup3.cml

Additional supplementary materials:  crystallographic information; 3D view; checkCIF report

## Figures and Tables

**Fig. 1.**
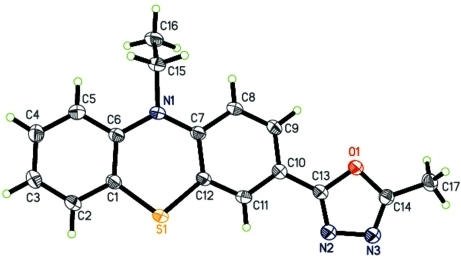
The molecular structure of the title compound with the atom-labelling scheme. Displacement ellipsoids are drawn at the 30% probability level. H atoms are represented as small spheres of arbitrary radii.

## supplementary crystallographic information

### Comment

The derivatives of phenothiazine are a series important chemical intermediates in design of the dye-sensitized solar cells (DSSCs) (Kim *et al.*, 2011; Hagfeldt *et al.*, 2010). As part of our interest in these materials, here we report the crystal structure of the title compound C_17_H_15_N_3_OS.

The title molecule is in a nonlplanar butterfly conformation with a dihedral angle of 13.68 (7)° between two benzene rings (Fig. 1). The crystal packing exhibits a π–π interaction with a centroid-centroid distance of 3.752 (2) Å between the benzene rings from the neighbouring molecules.

### Experimental

A solution of 5-[3-(10-ethyl)phenothiazyl]-tetrazole (500 mg, 1.69 mmol) in 10 ml acetic anhydride was heated to reflux and stirred for 1 h. The excess acetic anhydride was evaporated and the residue solid was extracted three times with dichloromethane. Then the organic layer was washed with water and dried with anhydrous sodium sulfate. After removal of the solvent, the crude product was purified by chromatography on a silica gel column using dichloromethane-ethyl acetate (*v*/*v* = 10:1) as eluent and isolated as a yellow powder. Yield: 472 mg (90%). The yellow single crystals suitable for X-ray diffraction were obtained after several days by slow evaporation of a mixture solution of dichloromethane and petroleum ether.

### Refinement

H atoms were placed in calculated positions (C—H = 0.93–0.97 Å) and treated as riding atoms, with *U*_iso_(H) = 1.2 or 1.5*U*_eq_(C).

### Crystal data

**Table:**

C_17_H_15_N_3_OS	*Z* = 2
*M**_r_* = 309.38	*F*(000) = 324
Triclinic, *P*1	*D*_x_ = 1.404 Mg m^−^^3^
Hall symbol: -P 1	Mo *K*α radiation, λ = 0.71073 Å
*a* = 7.6752 (4) Å	Cell parameters from 2708 reflections
*b* = 8.2913 (4) Å	θ = 3.0–31.6°
*c* = 12.9469 (8) Å	µ = 0.23 mm^−^^1^
α = 84.870 (4)°	*T* = 293 K
β = 82.569 (4)°	Block, yellow
γ = 63.696 (3)°	0.15 × 0.15 × 0.10 mm
*V* = 731.92 (7) Å^3^	

### Data collection

**Table:**

Bruker SMART APEX diffractometer	2554 independent reflections
Radiation source: fine-focus sealed tube	2217 reflections with *I* > 2σ(*I*)
Graphite monochromator	*R*_int_ = 0.020
φ and ω scans	θ_max_ = 25.0°, θ_min_ = 2.7°
Absorption correction: multi-scan (*SADABS*; Sheldrick, 2004)	*h* = −9→9
*T*_min_ = 0.968, *T*_max_ = 0.979	*k* = −9→9
5348 measured reflections	*l* = −15→12

### Refinement

**Table:**

Refinement on *F*^2^	Primary atom site location: structure-invariant direct methods
Least-squares matrix: full	Secondary atom site location: difference Fourier map
*R*[*F*^2^ > 2σ(*F*^2^)] = 0.039	Hydrogen site location: inferred from neighbouring sites
*wR*(*F*^2^) = 0.107	H-atom parameters constrained
*S* = 1.05	*w* = 1/[σ^2^(*F*_o_^2^) + (0.0547*P*)^2^ + 0.2148*P*] where *P* = (*F*_o_^2^ + 2*F*_c_^2^)/3
2554 reflections	(Δ/σ)_max_ < 0.001
201 parameters	Δρ_max_ = 0.33 e Å^−^^3^
0 restraints	Δρ_min_ = −0.28 e Å^−^^3^

### Special details

**Table:**

Geometry. All e.s.d.'s (except the e.s.d. in the dihedral angle between two l.s. planes) are estimated using the full covariance matrix. The cell e.s.d.'s are taken into account individually in the estimation of e.s.d.'s in distances, angles and torsion angles; correlations between e.s.d.'s in cell parameters are only used when they are defined by crystal symmetry. An approximate (isotropic) treatment of cell e.s.d.'s is used for estimating e.s.d.'s involving l.s. planes.
Refinement. Refinement of *F*^2^ against ALL reflections. The weighted *R*-factor *wR* and goodness of fit *S* are based on *F*^2^, conventional *R*-factors *R* are based on *F*, with *F* set to zero for negative *F*^2^. The threshold expression of *F*^2^ > σ(*F*^2^) is used only for calculating *R*-factors(gt) *etc*. and is not relevant to the choice of reflections for refinement. *R*-factors based on *F*^2^ are statistically about twice as large as those based on *F*, and *R*- factors based on ALL data will be even larger.

### Fractional atomic coordinates and isotropic or equivalent isotropic displacement parameters (Å^2^)

**Table:**

	*x*	*y*	*z*	*U*_iso_*/*U*_eq_	
S1	0.37243 (8)	0.17365 (6)	0.11098 (4)	0.05078 (19)	
O1	0.39396 (19)	0.69128 (15)	−0.27750 (9)	0.0430 (3)	
C12	0.2887 (2)	0.3831 (2)	0.04285 (13)	0.0353 (4)	
N1	0.0965 (2)	0.54971 (18)	0.19818 (11)	0.0410 (4)	
C10	0.3234 (3)	0.5423 (2)	−0.11819 (13)	0.0388 (4)	
C6	0.0893 (3)	0.3994 (2)	0.25572 (13)	0.0376 (4)	
C7	0.1642 (2)	0.5454 (2)	0.09210 (13)	0.0371 (4)	
C1	0.2039 (3)	0.2227 (2)	0.22245 (13)	0.0377 (4)	
N2	0.5266 (2)	0.3956 (2)	−0.27726 (12)	0.0460 (4)	
C13	0.4168 (3)	0.5350 (2)	−0.22376 (13)	0.0388 (4)	
C11	0.3653 (3)	0.3829 (2)	−0.05932 (13)	0.0382 (4)	
H11	0.4470	0.2736	−0.0898	0.046*	
C9	0.1961 (3)	0.7029 (2)	−0.07199 (14)	0.0452 (5)	
H9	0.1633	0.8110	−0.1103	0.054*	
C5	−0.0285 (3)	0.4198 (2)	0.34965 (14)	0.0450 (4)	
H5	−0.1070	0.5351	0.3734	0.054*	
C15	0.0098 (3)	0.7238 (2)	0.24919 (15)	0.0454 (5)	
H15A	0.0783	0.7936	0.2189	0.054*	
H15B	0.0307	0.7016	0.3224	0.054*	
N3	0.5841 (2)	0.4600 (2)	−0.37384 (12)	0.0477 (4)	
C8	0.1174 (3)	0.7040 (2)	0.03038 (14)	0.0453 (5)	
H8	0.0307	0.8133	0.0592	0.054*	
C2	0.1976 (3)	0.0770 (2)	0.28143 (15)	0.0462 (5)	
H2	0.2731	−0.0388	0.2577	0.055*	
C14	0.5034 (3)	0.6320 (2)	−0.36985 (13)	0.0424 (4)	
C4	−0.0320 (3)	0.2730 (3)	0.40882 (15)	0.0498 (5)	
H4	−0.1109	0.2908	0.4716	0.060*	
C16	−0.2063 (3)	0.8351 (3)	0.24062 (18)	0.0573 (6)	
H16A	−0.2300	0.8537	0.1685	0.086*	
H16B	−0.2488	0.9493	0.2713	0.086*	
H16C	−0.2773	0.7728	0.2766	0.086*	
C3	0.0812 (3)	0.1007 (3)	0.37469 (16)	0.0521 (5)	
H3	0.0790	0.0020	0.4140	0.063*	
C17	0.5164 (4)	0.7672 (3)	−0.44929 (15)	0.0558 (5)	
H17A	0.5952	0.8184	−0.4272	0.084*	
H17B	0.3878	0.8606	−0.4578	0.084*	
H17C	0.5748	0.7102	−0.5145	0.084*	

### Atomic displacement parameters (Å^2^)

**Table:**

	*U*^11^	*U*^22^	*U*^33^	*U*^12^	*U*^13^	*U*^23^
S1	0.0589 (3)	0.0257 (3)	0.0468 (3)	−0.0033 (2)	0.0067 (2)	0.00176 (18)
O1	0.0540 (8)	0.0336 (6)	0.0387 (7)	−0.0177 (6)	−0.0011 (6)	0.0001 (5)
C12	0.0363 (9)	0.0266 (8)	0.0403 (9)	−0.0112 (7)	−0.0049 (7)	0.0004 (7)
N1	0.0469 (9)	0.0259 (7)	0.0425 (8)	−0.0103 (6)	0.0023 (7)	−0.0035 (6)
C10	0.0405 (10)	0.0356 (9)	0.0403 (9)	−0.0169 (8)	−0.0050 (8)	0.0008 (7)
C6	0.0380 (9)	0.0298 (9)	0.0413 (9)	−0.0111 (7)	−0.0060 (7)	−0.0005 (7)
C7	0.0352 (9)	0.0287 (8)	0.0438 (9)	−0.0110 (7)	−0.0022 (7)	−0.0016 (7)
C1	0.0412 (10)	0.0304 (9)	0.0393 (9)	−0.0134 (7)	−0.0066 (7)	0.0008 (7)
N2	0.0514 (10)	0.0370 (8)	0.0437 (8)	−0.0154 (7)	−0.0002 (7)	0.0004 (7)
C13	0.0407 (10)	0.0325 (9)	0.0427 (9)	−0.0156 (8)	−0.0067 (8)	0.0023 (7)
C11	0.0393 (10)	0.0281 (8)	0.0430 (9)	−0.0103 (7)	−0.0039 (8)	−0.0048 (7)
C9	0.0492 (11)	0.0290 (9)	0.0492 (10)	−0.0119 (8)	−0.0024 (9)	0.0070 (8)
C5	0.0437 (10)	0.0377 (10)	0.0460 (10)	−0.0118 (8)	0.0002 (8)	−0.0035 (8)
C15	0.0516 (11)	0.0325 (9)	0.0502 (10)	−0.0170 (8)	−0.0002 (9)	−0.0068 (8)
N3	0.0526 (10)	0.0434 (9)	0.0423 (8)	−0.0181 (8)	0.0011 (7)	−0.0023 (7)
C8	0.0472 (11)	0.0268 (9)	0.0505 (11)	−0.0078 (8)	0.0020 (9)	−0.0011 (7)
C2	0.0545 (12)	0.0309 (9)	0.0496 (10)	−0.0158 (8)	−0.0053 (9)	0.0009 (8)
C14	0.0471 (11)	0.0425 (10)	0.0374 (9)	−0.0197 (8)	−0.0031 (8)	−0.0014 (7)
C4	0.0507 (12)	0.0497 (11)	0.0452 (10)	−0.0212 (10)	0.0014 (9)	0.0039 (9)
C16	0.0530 (12)	0.0355 (10)	0.0696 (13)	−0.0089 (9)	0.0043 (10)	−0.0056 (9)
C3	0.0612 (13)	0.0424 (10)	0.0523 (11)	−0.0249 (10)	−0.0033 (10)	0.0093 (9)
C17	0.0736 (15)	0.0516 (12)	0.0451 (11)	−0.0321 (11)	−0.0024 (10)	0.0049 (9)

### Geometric parameters (Å, º)

**Table:**

S1—C1	1.7541 (17)	C9—H9	0.9300
S1—C12	1.7583 (17)	C5—C4	1.388 (3)
O1—C14	1.362 (2)	C5—H5	0.9300
O1—C13	1.365 (2)	C15—C16	1.512 (3)
C12—C11	1.376 (2)	C15—H15A	0.9700
C12—C7	1.409 (2)	C15—H15B	0.9700
N1—C7	1.402 (2)	N3—C14	1.282 (2)
N1—C6	1.413 (2)	C8—H8	0.9300
N1—C15	1.472 (2)	C2—C3	1.379 (3)
C10—C9	1.387 (2)	C2—H2	0.9300
C10—C11	1.392 (2)	C14—C17	1.480 (2)
C10—C13	1.452 (2)	C4—C3	1.380 (3)
C6—C5	1.394 (2)	C4—H4	0.9300
C6—C1	1.407 (2)	C16—H16A	0.9600
C7—C8	1.402 (2)	C16—H16B	0.9600
C1—C2	1.387 (2)	C16—H16C	0.9600
N2—C13	1.287 (2)	C3—H3	0.9300
N2—N3	1.411 (2)	C17—H17A	0.9600
C11—H11	0.9300	C17—H17B	0.9600
C9—C8	1.383 (2)	C17—H17C	0.9600
			
C1—S1—C12	101.74 (8)	N1—C15—H15A	108.5
C14—O1—C13	102.81 (13)	C16—C15—H15A	108.5
C11—C12—C7	121.19 (15)	N1—C15—H15B	108.5
C11—C12—S1	116.40 (12)	C16—C15—H15B	108.5
C7—C12—S1	122.15 (13)	H15A—C15—H15B	107.5
C7—N1—C6	123.14 (14)	C14—N3—N2	106.14 (14)
C7—N1—C15	118.30 (14)	C9—C8—C7	122.01 (16)
C6—N1—C15	118.23 (14)	C9—C8—H8	119.0
C9—C10—C11	118.04 (16)	C7—C8—H8	119.0
C9—C10—C13	122.68 (16)	C3—C2—C1	121.15 (17)
C11—C10—C13	119.26 (15)	C3—C2—H2	119.4
C5—C6—C1	117.04 (16)	C1—C2—H2	119.4
C5—C6—N1	121.22 (15)	N3—C14—O1	112.57 (15)
C1—C6—N1	121.73 (15)	N3—C14—C17	129.09 (17)
C8—C7—N1	121.38 (15)	O1—C14—C17	118.33 (16)
C8—C7—C12	116.46 (15)	C3—C4—C5	120.19 (17)
N1—C7—C12	122.11 (15)	C3—C4—H4	119.9
C2—C1—C6	120.68 (16)	C5—C4—H4	119.9
C2—C1—S1	116.70 (13)	C15—C16—H16A	109.5
C6—C1—S1	122.48 (13)	C15—C16—H16B	109.5
C13—N2—N3	106.45 (14)	H16A—C16—H16B	109.5
N2—C13—O1	112.02 (15)	C15—C16—H16C	109.5
N2—C13—C10	128.50 (16)	H16A—C16—H16C	109.5
O1—C13—C10	119.47 (15)	H16B—C16—H16C	109.5
C12—C11—C10	121.51 (15)	C2—C3—C4	119.06 (17)
C12—C11—H11	119.2	C2—C3—H3	120.5
C10—C11—H11	119.2	C4—C3—H3	120.5
C8—C9—C10	120.70 (16)	C14—C17—H17A	109.5
C8—C9—H9	119.6	C14—C17—H17B	109.5
C10—C9—H9	119.6	H17A—C17—H17B	109.5
C4—C5—C6	121.87 (17)	C14—C17—H17C	109.5
C4—C5—H5	119.1	H17A—C17—H17C	109.5
C6—C5—H5	119.1	H17B—C17—H17C	109.5
N1—C15—C16	114.91 (16)		
